# Phosphorylation as a candidate regulatory mechanism for effector recruitment to tankyrase

**DOI:** 10.1098/rsos.250824

**Published:** 2025-10-15

**Authors:** Benjamin J. Broadway, Katie Pollock, Nora Cronin, Robert Rottapel, Frank Sicheri, Sebastian Guettler

**Affiliations:** ^1^ Division of Structural Biology, The Institute of Cancer Research, London, UK; ^2^ Division of Cell and Molecular Biology, The Institute of Cancer Research, London, UK; ^3^ Division of Cancer Therapeutics, The Institute of Cancer Research, London, UK; ^4^ Princess Margaret Cancer Centre, University Health Network, University of Toronto, Toronto, Ontario, Canada; ^5^ Departments of Medicine, Medical Biophysics and Immunology, University of Toronto, Toronto, Ontario, Canada; ^6^ Division of Rheumatology, St Michael's Hospital, Toronto, Ontario, Canada; ^7^ Lunenfeld-Tanenbaum Research Institute, Mount Sinai Hospital, Toronto, Ontario, Canada; ^8^ Departments of Molecular Genetics and Biochemistry, University of Toronto, Toronto, Ontario, Canada

**Keywords:** signal transduction, tankyrase, MDC1, NUMA1, post-translational modification, ADP-ribosylation, phosphorylation, protein–protein interaction, X-ray crystallography, biochemistry

## Abstract

The ADP-ribosyltransferase tankyrase (with two paralogues, TNKS and TNKS2) plays pivotal roles in diverse cellular processes that encompass signal transduction, including Wnt/β-catenin, Hippo and toll-like receptor (TLR) signalling, mitotic spindle assembly, glucose homeostasis and telomere maintenance, among many other functions. Tankyrase recruits its effectors (substrates and binders) via a degenerate tankyrase-binding motif (TBM) and exerts its activities by subsequent substrate ADP-ribosylation and/or scaffolding. Variants of the TBM, found in diverse proteins, engage the ankyrin repeat cluster (ARC) domains of tankyrase. Yet, whether effector recruitment to tankyrase can be regulated has remained unknown. In this study, we propose that phosphorylation at position eight of the TBM enhances the affinity of effectors for the ARC domains of tankyrase. Using isolated TBM peptides, we demonstrate that phosphorylation of serine, but not tyrosine, strengthens ARC binding by up to an order of magnitude. Interrogation of proteome-wide phosphorylation data reveals that phosphorylation at position eight in the TBM is enriched in proteins that support centrosome function/localization. Our findings suggest that TBM phosphorylation may serve as an effector-specific mechanism for tankyrase recruitment/retention, providing an additional layer of regulation to control tankyrase.

## 1. Introduction

Tankyrase (with two paralogues: TNKS and TNKS2) is part of the Diphtheria-toxin like family of ADP-ribosyltransferases (ARTDs) [1]. Catalytically active members of this family either transfer a single unit of ADP-ribose from their co-substrate NAD^+^ onto acceptor proteins, resulting in mono(ADP-ribosyl)ation (MARylation), or synthesize ADP-ribose chains, giving rise to substrate poly-ADP-ribosylation (PARylation) [2]. Diverse cellular functions of tankyrase have been reported [3,4]; these include mitotic resolution and elongation of telomeres [5–9], Wnt/β-catenin signalling [10–13], Hippo signalling [14–16], DNA repair by homologous recombination (HR) [17], glucose metabolism [18], centrosome function [19], macrophage activation, bone turnover, toll-like receptor (TLR) signalling [20,21] and epithelial lumen formation [22]. These varied biological roles imply that tankyrase may be regulated in a pathway-specific manner, allowing contextual control of this otherwise seemingly pleiotropic protein.

**Figure 1.**
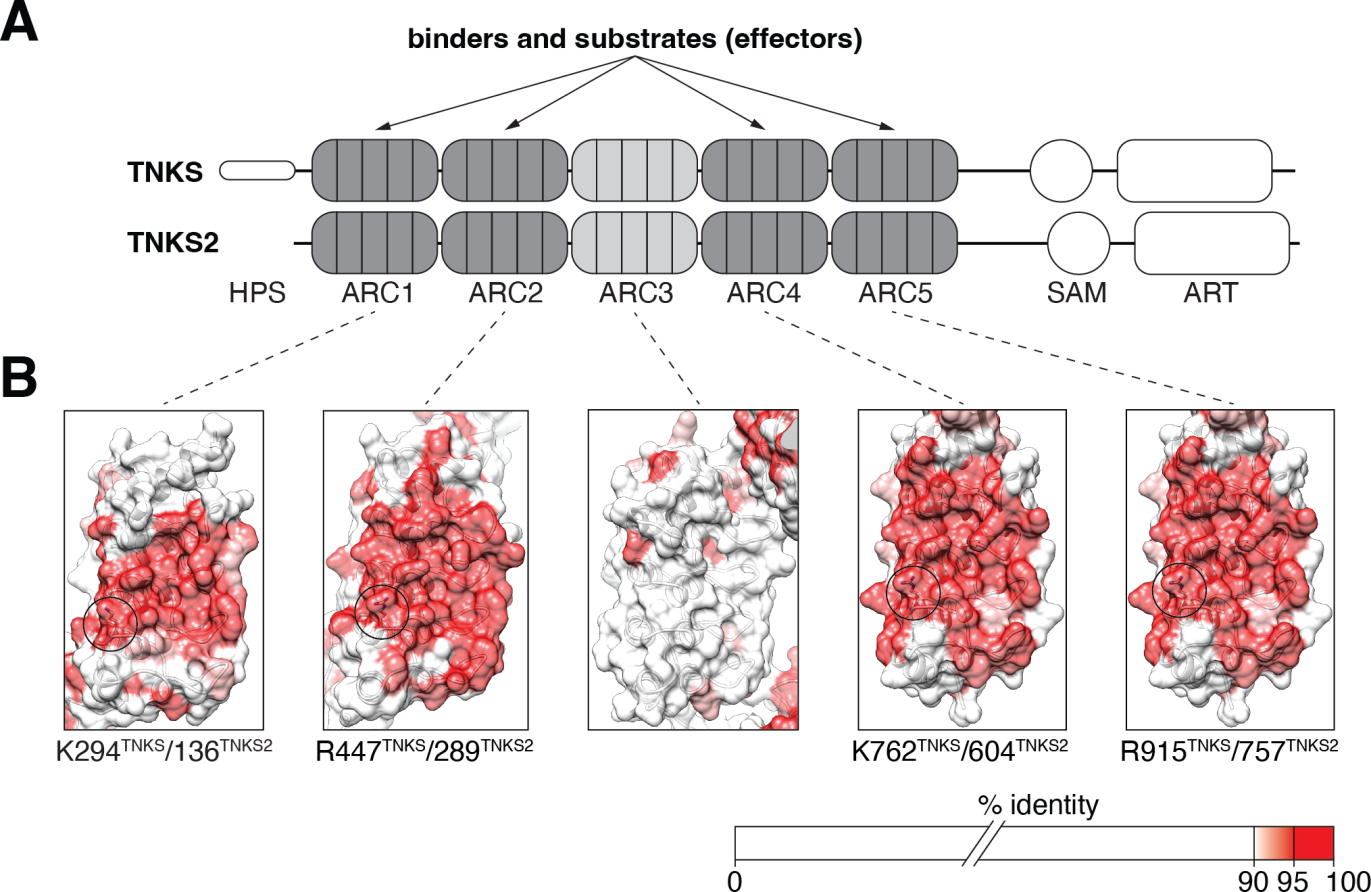
Tankyrase domain organization and conserved phospho-recognition infrastructure of tankyrase ARCs. (A) Schematic representation of human TNKS and TNKS2. (B) Structural representations of human TNKS ARCs, obtained from the AlphaFold Protein Structure Database (accession number AF-O95271-F1) [23,24], in cartoon representation with transparent surfaces coloured by sequence conservation. The pS8-recognizing residues are circled. See §5 for accession numbers of protein sequences used for the multiple sequence alignment to assess conservation.

Tankyrase contains five ankyrin repeat clusters (ARCs), a sterile alpha motif (SAM) domain and a C-terminal catalytic ADP-ribosyltransferase (ART) domain with PARylation capability (figure 1A) [3,25]. The ARC modules form a platform that enables interactors to be recruited. Among these, substrates are directed to the catalytic ART domain for PARylation, but not all effectors are modified in this manner [26–29]. Four of the five ARCs interact with tankyrase-binding motifs (TBMs), namely ARCs 1, 2, 4 and 5; ARC3 does not feature the TBM-binding infrastructure [27–29]. A shared characteristic of nearly all known tankyrase binders is the presence of a degenerate six- to eight-amino-acid TBM of the consensus sequence R-x-x-[small hydrophobic or G]-[D/E]-G-[no P]-[D/E] [28,30]. An acidic amino acid at position eight increases the affinity of the TBM for tankyrase and can compensate for suboptimal amino acids at other positions [28]. Conversely, basic residues at position eight are the least preferred [28]. Consistent with this, intracellular TBM deep mutational scanning demonstrated a preference for TBMs containing acidic amino acids at position eight, and to a lesser extent at positions nine and ten, for binding TNKS2 ARC4 [31]. Basic residues at position eight were disfavoured [31].

*In silico* prediction and proteomics approaches have identified a vast number of structurally and functionally diverse tankyrase binding partners, some of which undergo ADP-ribosylation while others do not [22,28,32–34]. This aligns with the observed catalysis-independent functions of tankyrase as a scaffolding protein [11,17,33]. The mere presence of a TBM alone does not indicate whether a tankyrase binder is also PARylated, suggesting that there are substrate-intrinsic determinants [35]. Given the high prevalence of the TBM across the proteome, it is difficult to ascertain how specific signalling can be achieved. One potential mechanism for attaining target specificity involves the high local concentrations of particular tankyrase effectors at certain subcellular sites, for example the mitotic spindle poles or β-catenin signalling complexes [5,36]. A second mechanism could arise from the multimerization of TBM-bearing proteins, which effectively is equivalent to multiple occurrences of TBMs in the same polypeptide chain [25]. Beyond these possibilities, we do not know of any regulatory events that could direct tankyrase to specific targets through TBM–ARC interactions.

Reversible protein phosphorylation is a key post-translational modification that governs most cellular processes [37]. Given the contribution of acidic residues at TBM position eight towards TBM affinity for ARCs [28,31], we hypothesized that phosphorylation at this position may produce an effect comparable with an acidic residue, offering a novel means to regulate tankyrase in a context-dependent manner.

The mediator of DNA damage checkpoint protein 1 (MDC1) is a critical scaffold that acts in both the HR and non-homologous end joining pathways for DNA damage repair [38,39] and mitotic progression [40,41]. Tankyrase recognizes two TBMs in MDC1 to support DNA damage repair [17]. We noticed that one of the identified TBMs, ^947^RGEPEGGS^955^, is reported to be phosphorylated at position eight in cultured cells [42]. We used MDC1 as a model protein to interrogate potential phospho-regulation of effector recruitment to tankyrase. Using peptide binding assays and X-ray crystallography, we show that TBM phosphorylation of serine at position eight enhances TBM binding several-fold. We confirm this using additional TBMs from other tankyrase effectors, demonstrating that the equivalent phosphorylation of tyrosine does not enhance TBM binding. By interrogating existing phosphorylation data, we curate phosphorylation events at TBM position eight across the human proteome and suggest that this mechanism may be widely employed in various contexts of tankyrase function.

## 2. Results

### 2.1. Phosphorylation of an MDC1 tankyrase-binding motif enhances its affinity for tankyrase

**Figure 2.**
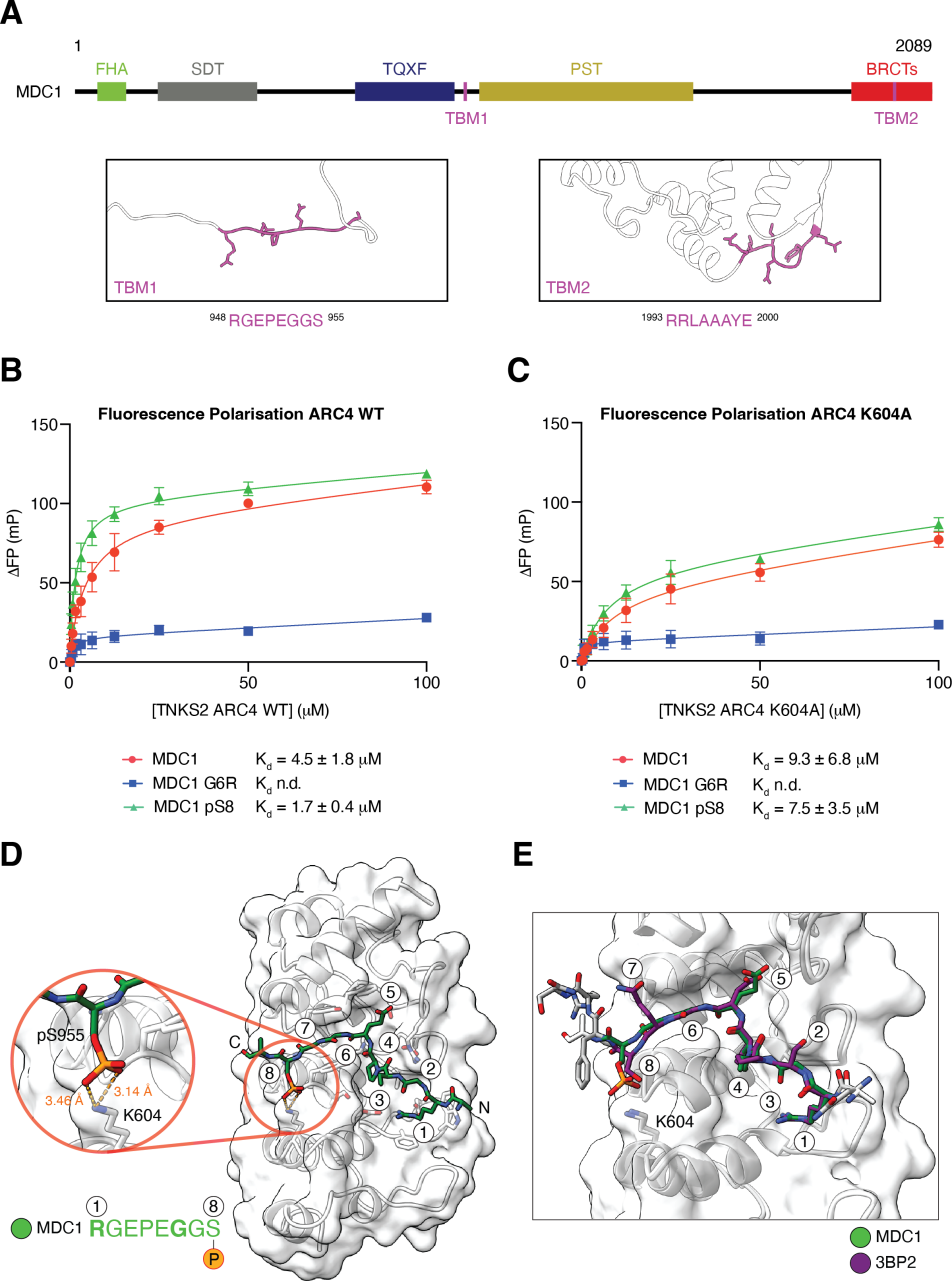
MDC1 TBM1 position-eight serine phosphorylation increases affinity for tankyrase. (A) Schematic representation of MDC1 domain organization. The structural model of MDC1 (Uniprot ID: Q14676) was retrieved from the AlphaFold Protein Structure Database (accession number AF-Q14676-F1-v4) [23,24]. Regions surrounding the two TBMs are shown in white cartoon representation while the TBM side chains are shown in purple stick representation. (B) and (C) Fluorescence polarization (FP) assays measuring binding of the indicated fluorescently labelled MDC1 TBM1 peptides to TNKS2 ARC4 in its wild-type (B) and K604A mutant (C) forms. ΔFP values denote the change in millipolarization units relative to baseline conditions without protein. Data points are means ± standard error of the mean (s.e.m.) from *n* = 3 separate experiments that were performed in technical duplicate. Dissociation constants (*K*_*d*_), with standard errors of the fit, were obtained by nonlinear regression with a one-site total binding model. n.d. = not determined. (D) Structural representation of TNKS2 ARC4 (in cartoon and transparent surface representation with K604 shown as sticks) bound to an MDC1 TBM1 peptide corresponding to the sequence ^943^ERDTQRGEPEGGpSQDQ^958^, in green stick representation, coloured by heteroatom. The octapeptide residues of the MDC1 TBM are numbered. The inset shows a salt bridge between pS955 at position eight of the MDC1 TBM and K604 of TNKS2 ARC4. (E) Superimposition of TNKS2 ARC4 complexes with the phospho-MDC1 or 3BP2 (3TWR) [28] TBM peptides. Phospho-MDC1 and 3BP2 octapeptides are shown in stick representation and coloured as indicated and by heteroatom, with TBM positions numbered and corresponding ARC4 chains shown in grey and white, respectively. The transparent white ARC4 surface corresponds to the phospho-MDC1 complex. Peptide residues outside the TBM octapeptides are shown in grey and white.

MDC1 has an N-terminal forkhead-associated (FHA) domain, two BRCA1 C-terminal (BRCT) domains and is otherwise predicted to be largely disordered (figure 2A) [23]. MDC1 contains two TBMs which both contribute to tankyrase binding and co-localization of MDC1 and tankyrase at DNA damage sites [17]. The more N-terminal motif (TBM1, amino acids 948−955) resides within a disordered region of the protein and bears a serine residue at position eight (figure 2A). TBM1 is well-conserved across mammals, including rodents, but noticeably absent in reptiles and fish (electronic supplementary material, figure S1A). The more C-terminal TBM (TBM2, amino acids 1993−2000), which maps to a loop within the tandem BRCT domains (figure 2A) [23], has an acidic amino acid at position eight (electronic supplementary material, figure S1A).

To test if phosphorylation of the N-terminal motif enhances tankyrase binding, we performed fluorescence polarization (FP) assays with fluorescently labelled MDC1 TBM1 peptides and TNKS2 ARC4 as a representative ARC. Non-phosphorylated TBM1 bound ARC4 with a *K*_*d*_ of 4.5 ± 1.8 µM. TBM1 phosphorylation at position eight reproducibly increased ARC4 affinity approximately threefold (*K*_*d*_ = 1.7 ± 0.4 µM; figure 2B). As expected [28], mutation of glycine at position six to arginine (G6R) abolished measurable binding to the ARC (figure 2B). To predict the protein kinases capable of phosphorylating this site in cells, we conducted a kinase search, interrogating a substrate specificity atlas for the human serine/threonine kinome [43]. This analysis identified ataxia-telangiectasia mutated (ATM) and ataxia telangiectasia and Rad3-related (ATR) as the third- and sixth-highest ranking kinases for this site, respectively (electronic supplementary material, figure S1B).

How does phosphorylation at position eight increase TBM affinity? A hint was provided by a previously determined crystal structure of TNKS2 ARC4 bound to a TBM from the tankyrase binder myeloid cell leukaemia sequence 1 (MCL1) [28]. In this structure, an acidic side chain, glutamate, at position eight in the TBM forms a salt bridge with a basic residue, K604, in ARC4 [28]. We thus repeated the FP assay using MDC1 TBM1 phosphovariants and TNKS2 ARC4 bearing the K604A mutation. The interaction gain conferred by MDC1 TBM1 position-eight phosphorylation was attenuated by the K604A mutation as both the non-phosphorylated and phosphorylated peptides bound the ARC4 K604A variant with similar affinities, with *K*_*d*_ values of 9.3 ± 6.8 and 7.5 ± 3.5 µM, respectively (figure 2C).

We obtained similar results for the TBM of the tankyrase substrate 3BP2, a well-studied model substrate [28]. Proteomics data indicate that the 3BP2 TBM is phosphorylated at position two [44]. Although there is no evidence for position-eight phosphorylation, we assessed binding of the unphosphorylated 3BP2 TBM as well as single- and dual-site modified peptides with phosphorylations at positions two and eight, to explore if phosphoregulation applies more generally (electronic supplementary material, figure S2). The non-phosphorylated 3BP2 TBM showed similar affinities for WT and K604A ARC4 (*K*_*d*_ = 5.5 ± 0.3 and 3.8 ± 0.2 µM, respectively) (electronic supplementary material, figure S2A,B). However, phosphorylation at position eight substantially enhanced binding to WT ARC4 (*K*_*d*_ = 0.4 ± 0.04 µM), by an order of magnitude. This enhancement was lost with the K604A mutant variant (*K*_*d*_ = 2.0 ± 0.1 µM) (electronic supplementary material, figure S2A,B).

The general ARC4-MDC1 TBM1 affinity drop, irrespective of peptide phosphorylation status, upon introduction of the K604A mutation (figure 2B,C) may be explained by weak hydrogen bonding between the unphosphorylated serine at position eight of MDC1 TBM1 and K604. Distances compatible with hydrogen bonding were observed in two out of four ARC4-TBM peptide complexes in the asymmetric unit in a co-crystal structure of TNKS2 ARC4 bound to the 3BP2 TBM [28], although in the specific case of 3BP2, K604 does not appear to contribute to the binding affinity of the unphosphorylated TBM for TNKS2 ARC4 as measured by FP (electronic supplementary material, figure S2).

Collectively, these results indicate that TNKS2 K604 is critical for recognizing the phosphorylated serine at position eight in TBM peptides from both MDC1 and 3BP2.

### 2.2. Structural basis for tankyrase-binding motif position-eight phospho-serine recognition by tankyrase

To confirm the underlying molecular mechanism of phospho-regulated binding, we co-crystallized TNKS2 ARC4 with an MDC1 TBM1 peptide phosphorylated at position eight of the motif (^943^ERDTQRGEPEGGpS*QDQ^958^) and solved the structure by molecular replacement (table 1; see §5). The asymmetric unit consists of two ARC-peptide complexes, which are highly similar (electronic supplementary material, figure S3). The TBM binds ARC4 in the previously observed binding mode [28], engaging a well-defined pocket consisting of a cradle that accommodates the arginine residue at position one, a central hydrophobic region that engages proline at position four, and an aromatic channel that sandwiches glycine at position six (figure 2D). This binding mode is essentially identical to that of 3BP2 and numerous other tankyrase binders [28] (figure 2E). The phosphorylated serine residue at position eight establishes a salt bridge with TNKS2 K604 (figure 2D). This aligns with our FP binding data showing the importance of K604 for phospho-recognition (figure 2B). The peptide chain is slightly displaced (by approx. 1.3 Å at the Cα of serine at position eight) due to salt bridge formation, which helps accommodate the bulky phosphate group (figure 2E). There are otherwise no substantial phosphorylation-induced structural rearrangements within either the MDC1 TBM or TNKS2 ARC4. A nearby K602 side chain does not read out the phospho-serine modification in the presence of K604 (electronic supplementary material, figure S3).

**Table 1.** Data collection and refinement statistics for TNKS2 ARC4-MDC1 phospho-TBM1.

data collection^a^	
PDB ID	9QJZ
beamline	Diamond I04
wavelength (Å)	0.97795
space group	P6_5_
unit cell a, b, c (Å)	118.96, 118.96, 48.32
unit cell α, β, γ (°)	90, 90, 120
molecules/ASU	2
resolution (Å)^a^^,b^	29.74–2.31 (2.39–2.31)
total number of reflections^b^	175 378
number of unique reflections^b^	17 376
*R*_merge_ (all I+ and I−)^b^	0.787 (5.785)
*R*_meas_ (all I+ and I−)^b^	0.829 (6.129)
mean I/σI^b^	7.0 (1.5)
CC_1/2_^b^	0.941 (0.319)
completeness (%)^b^	100 (100)
multiplicity^b^	10.1 (9.2)
Wilson B factor (Å^2^)	33.79

refinement^a^	
resolution (Å)	29.74–2.31
*R*_work_/*R*_free_ (test set 5%)	0.185/0.225
reflections used in refinement	16 956
reflections in *R*_free_ test set	860
RMSD bond lengths (Å)	0.005
RMSD bond angles (°)	0.722
number of non-hydrogen protein atoms	2587
number of non-hydrogen solvent atoms	57
B factor protein (Å^2^)	39.62
B factor solvent (Å^2^)	38.10
Ramachandran favoured (%)	98.46
Ramachandran allowed (%)	1.54
Ramachandran disallowed (%)	0
rotamer outliers (%)	0.79

^a^ Values for the highest-resolution shell are shown in parentheses.

^b^ As calculated in AIMLESS.

Multiple sequence alignment revealed that a positively charged residue equivalent to K604, lysine in ARCs 1 and 4 and arginine in ARCs 2 and 5 (figure 1B), is essentially invariant across a wide range of phyla, from sponges to humans. The only point of deviation in our phylogenetic analysis occurs in the nematode *Toxocara canis*, where lysine is replaced by arginine, still preserving the charge [45].

In summary, we present compelling evidence for a conserved phospho-recognition infrastructure in tankyrase’s effector-binding ARCs.

### 2.3. Phosphorylation site mapping data highlight the prevalence of tankyrase-binding motif position-eight serine/threonine phosphorylation

To explore the prevalence of phospho-regulation of TBMs in the human proteome, we performed an *in silico* search for experimentally mapped phosphorylation events at position eight within TBMs (see §5 for details). To capture a comprehensive set of TBMs, we applied our analysis to a set of previously identified and predicted TBMs, ranked on the basis of their conformance to an experimentally defined TBM consensus sequence [28]. By interrogating PhosphoSite Plus [44], we found instances of TBM position-eight phosphorylation on 311 serine and 77 threonine residues (electronic supplementary material, table S1). We also identified 54 instances of tyrosine phosphorylation at this position (electronic supplementary material, table S1). While phospho-serine and phospho-threonine are compatible with salt bridge formation, phospho-tyrosine is less likely to support salt bridge formation due to its larger side chain (as further discussed below). Furthermore, our analysis recovered the MDC1 TBM1 octapeptide as the 21st-highest ranking TBM showing an experimentally observed phosphorylation.

### 2.4. Gene ontology enrichment analysis of proteins with serine/threonine phosphorylation at tankyrase-binding motif position eight predicts functions in centrosome biology

**Figure 3.**
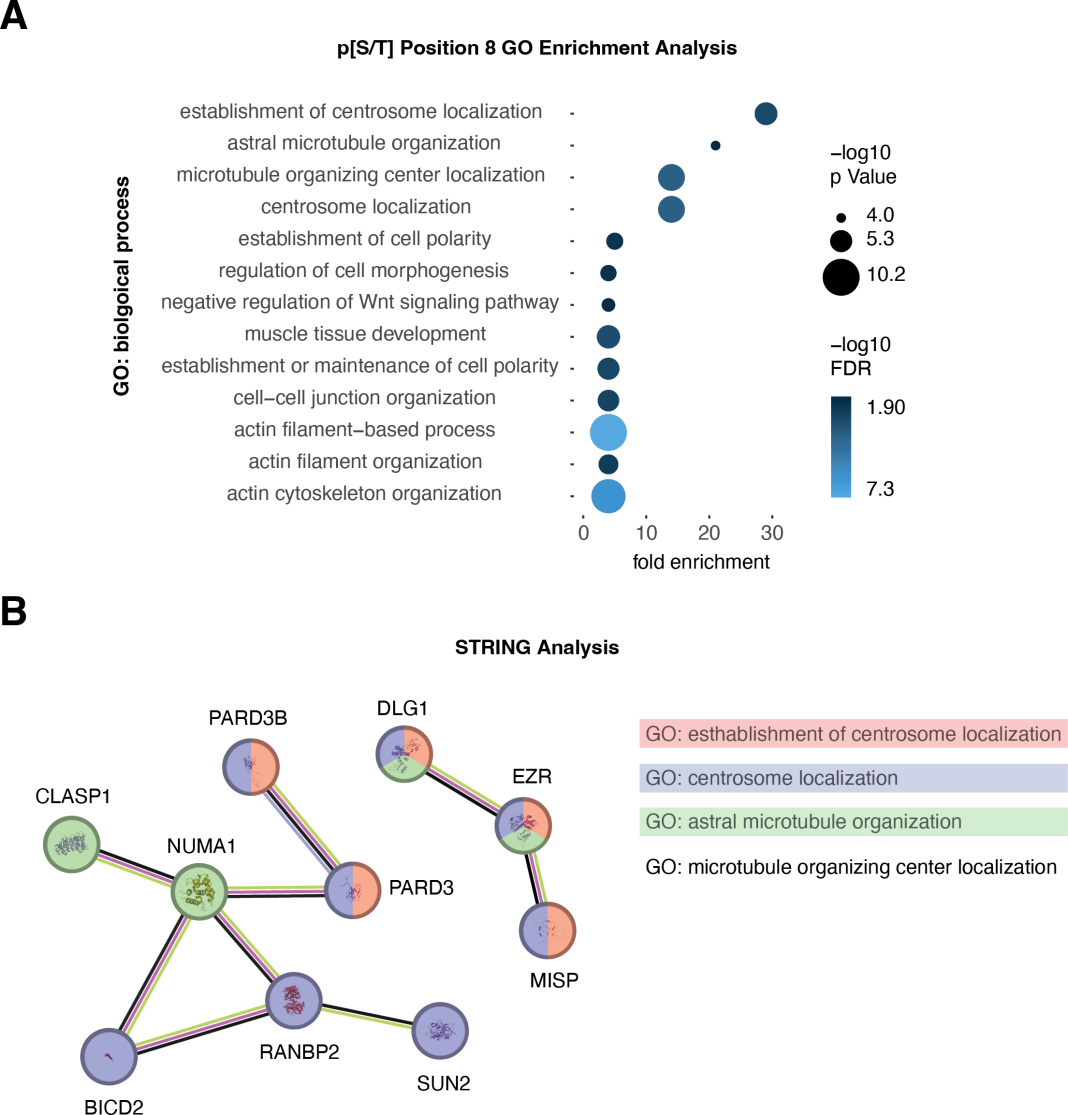
Gene set enrichment analysis suggests centrosome function is regulated by tankyrase effectors with S/T phosphorylation at TBM position eight. (A) The top 13 enriched gene ontology (GO) terms for biological processes from the 388 proteins with a TBM containing p[S/T] at position eight (date: 23 October 2023) [46]. *p*-value and false discovery rates are displayed as −log10 values shown in circles and blue shading, respectively. Only GOs with a −log10 (*p*-value) > 4 and at least fourfold enrichment are displayed. (B) STRING analysis [47] from the top four most highly enriched GO terms in (A). The lines between nodes denote an experimentally determined link (purple), conserved topological neighbourhood (green), co-expression (black) and shared protein homology (blue). Nodes, corresponding to proteins, are colour-coded by GO term. Note that “GO: microtubule organizing centre localization” is not represented in STRING.

To gain insights into the biological processes regulated by TBM phosphorylation, we performed a gene ontology (GO) enrichment analysis on proteins containing TBM octapeptides with phospho-serine or phospho-threonine at position eight (p[S/T]8). Significantly enriched biological processes (with a −log10(*p*-value) > 4 and fold enrichment > 4) included astral microtubule organization, negative regulation of the Wnt/β-catenin signalling pathway, and cell–cell junction organization, among others (figure 3A). Interestingly, the four most highly enriched GO terms were all related to centrosome biology (figure 3A). In line with this, several studies have highlighted important roles of tankyrase at the centrosome and mitotic spindle poles [5,19,48]. We next interrogated the Search Tool for the Retrieval of INteracting Genes/proteins (STRING) database [47], using the top four gene ontologies as queries, and observed a cluster with multiple interactions centred around the nuclear mitotic apparatus 1 (NUMA1) protein (figure 3B). NUMA1 itself is a reported tankyrase binder and substrate [30,49,50]. In mitosis, alongside its partner dynein, NUMA1 links the proteinaceous spindle poles to spindle microtubules [51] and co-localizes with tankyrase [30,49,50]. The tankyrase-NUMA1 relationship prompted us to investigate whether NUMA1 TBM phosphorylation could alter its affinity for tankyrase.

### 2.5. NUMA1 tankyrase-binding motif position-eight phosphorylation enhances its affinity for tankyrase

**Figure 4.**
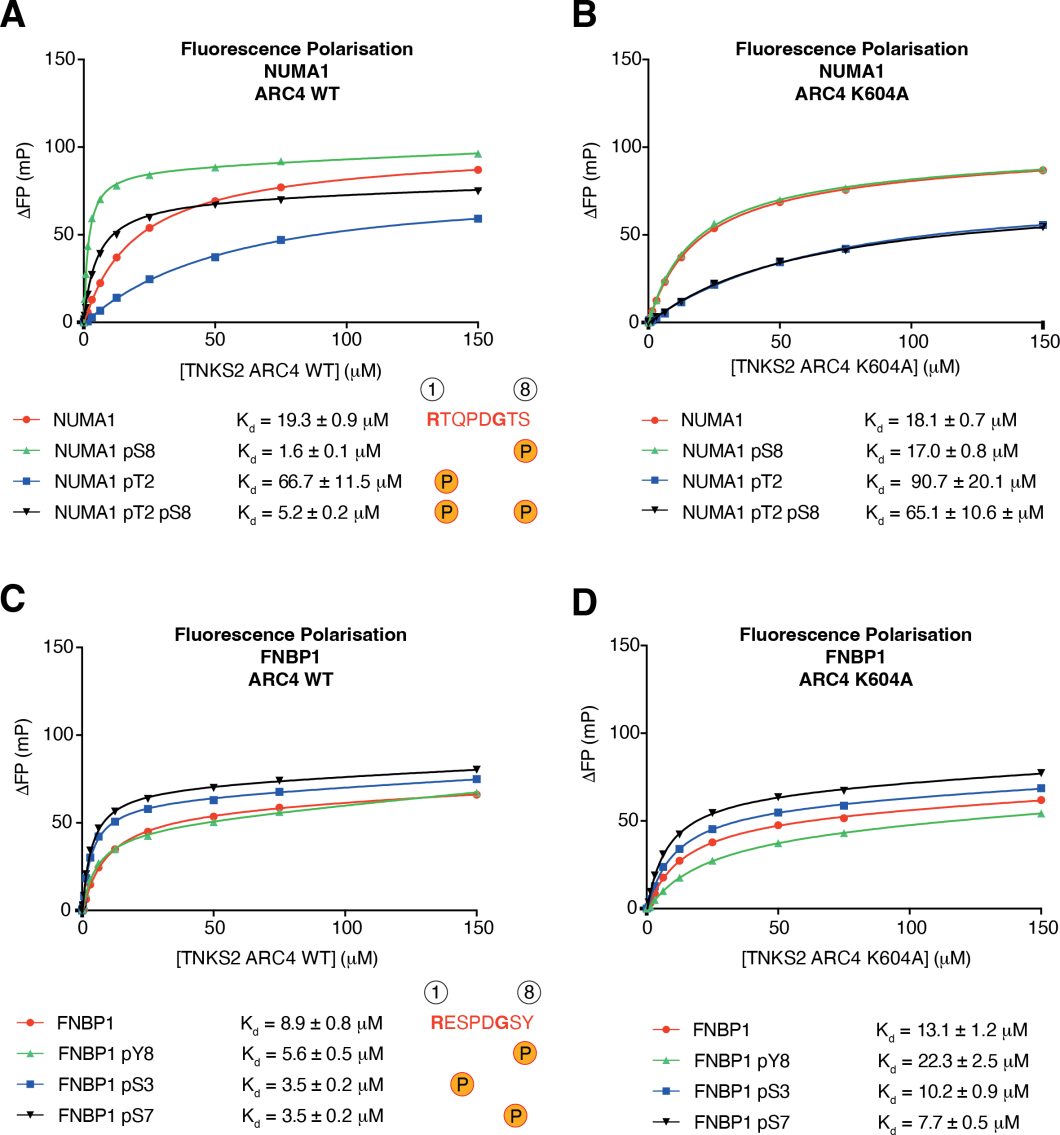
NUMA1 serine, but not FNBP1 tyrosine, TBM phosphorylation at position eight increases affinity for tankyrase. (A) and (B) FP assays for the indicated NUMA1 peptides and TNKS2 ARC4 variants, as in figure 2. A single experiment (*n* = 1) was performed in technical duplicate. Both data points are shown but may appear overlapping due to their similarity. Dissociation constants (*K*_*d*_), with standard errors of the fit, were obtained by nonlinear regression with a one-site total binding model. (C) and (D) FP assays as in (A), but for indicated FNBP1 TBM peptides.

Our *in silico* analysis uncovered that the NUMA1 TBM ^1743^RTQPDGTS^1750^ can be phosphorylated at positions two and eight (electronic supplementary material, table S1). To experimentally test if phosphorylation of this motif modulates tankyrase binding, we performed FP assays with either the unmodified NUMA1 TBM octapeptide, or with peptides phosphorylated at positions two (pT2) or eight (pS8). Phosphorylation at position eight increased TBM peptide affinity from a *K*_*d*_ of 19.3 ± 0.9 to 1.6 ± 0.1 μM (12-fold; figure 4A). Conversely, phosphorylation at position two gave rise to a moderate (approx. threefold) decrease in affinity (*K*_*d*_ = 66.7 ± 11.5 μM; figure 4A). As for MDC1 and 3BP2, FP assays with the ARC4 K604A mutant variant showed that NUMA1 pS8 and non-phospho TBM peptides bound the ARC with similar affinities (*K*_*d*_ = 17.0 ± 0.8 and 18.1 ± 0.7 μM, respectively; figure 4B). A NUMA1 pT2 and pS8 combination TBM peptide exhibited an increased ARC4 affinity (5.2 ± 0.2 μM) compared with the unmodified TBM peptide, but only in the context of wild-type ARC4 (figure 4B).

### 2.6. Tyrosine phosphorylation at tankyrase-binding motif position eight does not increase affinity for tankyrase

We identified 54 instances of tyrosine phosphorylation at position eight within TBMs (electronic supplementary material, table S1), raising the question of whether tyrosine phosphorylation can regulate the TBM–ARC interaction. To explore this, we focused on the TBM (^515^RESPDGSY^522^) of Formin binding protein 1 (FNBP1, also known as FBP17), for which our *in silico* search identified phosphorylation at positions three (pS3), seven (pS7) and eight (pY8). FNBP1 has been shown to facilitate the formation of cellular leading edges by detecting plasma membrane tension and promoting actin polymerization [52]. We performed FP assays with different phospho-variants of the FNBP1 TBM peptide. Unlike for pS8, pY8 did not substantially enhance the ARC4 affinity (approx. 1.6-fold, from 8.9 ± 0.8 to 5.6 ± 0.5 μM; figure 4C). Compared with the threefold increase in affinity upon MDC1 TBM1 S8 phosphorylation (figure 2B), 14-fold for 3BP2 (electronic supplementary material, figure S2A), and 12-fold for NUMA1 (figure 4A), this is a modest change. Likewise, the pS3 and pS7 FNBP1 TBM peptides had similar affinities for the ARC compared with the non-phosphorylated TBM peptide (figure 4C). The K604A mutation generally weakened ARC4 affinities for all FNBP1 TBM variants, suggesting that K604 may make contacts irrespective of TBM phosphorylation status. However, an approximately fourfold affinity drop for the pY8 peptide upon mutation of K604 (from 5.6 ± 0.5 to 22.3 ± 2.5 μM; figure 4C,D), which was larger than that observed for the other peptides, indicates that a pY8 contact with K604 may still contribute to tankyrase binding.

## 3. Discussion

In this study, we show that phosphorylation of serine, and most likely threonine (given their structural similarity), but not tyrosine, at position eight of TBMs enhances binding to an ARC domain of tankyrase by up to an order of magnitude. We demonstrate that phospho-recognition occurs through a basic residue that is highly conserved within all four effector-binding ARCs, making it part of the core TBM binding infrastructure (figure 1). Given the multivalency of TBM interactions across multiple ARCs [27,28] and the polymeric nature of tankyrase [11,45,53,54], the effective affinity (avidity) gain upon TBM position eight serine/threonine phosphorylation is probably even stronger than that which we have measured with a single ARC. This may particularly be the case where tankyrase effectors bear multiple TBMs or occur as multimers, further adding to avidity effects. We propose that TBM phosphorylation is a widespread mechanism for stabilizing tankyrase-effector complexes to regulate diverse cellular events. Control of tankyrase function at the effector level, rather than the tankyrase level, offers opportunities for context-specific tankyrase regulation. Our study further highlights the importance of considering a subset of TBMs as octapeptides rather than hexapeptides. Even in the absence of phospho-regulation, acidic amino acids at TBM position eight can strongly contribute to tankyrase binding [28].

### 3.1. MDC1 and tankyrase in the DNA damage response

Human MDC1 recruits tankyrase to sites of DNA damage, which promotes DNA damage repair by HR [17]. We chose to focus on an MDC1 peptide as a model, given that numerous mass spectrometry studies have shown that MDC1 TBM1 phosphorylation at position eight occurs in cultured cells [44]. Moreover, small-molecule inhibitors of the DNA damage response kinases ATM and ATR modulate this phosphorylation event in cells [42]. In line with this, our kinase prediction analysis ranks ATM and ATR among the top 10 candidates to phosphorylate this site, alongside other PIKK family kinases (electronic supplementary material, figure S1A). Thus, it is plausible that phosphorylation of MDC1 TBM1 at position eight could drive the recruitment/retention of tankyrase to/at foci of MDC1, provided the system is sensitive to the avidity gain. We hypothesize that the cell uses this regulatory mechanism to cluster tankyrase at damaged sites. Interestingly, tankyrase recruitment and subsequent DNA damage repair by HR do not appear to depend on tankyrase’s catalytic activity [17]. A similar scaffolding role has also been observed in the Wnt/β-catenin signalling pathway [11] and pexophagy [33]. Therefore, clustering of tankyrase may be required to recruit additional factors to MDC1-containing complexes to coordinate the DNA damage response. MDC1 phosphorylation may also tune other MDC1 functions beyond the DNA damage response [40,41].

### 3.2. Tankyrase localization to centrosomes and mitotic spindle poles

Our GO analysis of TBMs phosphorylated on serines/threonines at position eight reveals an enrichment of biological processes linked to various aspects of centrosome localization and function (figure 3A). This includes astral microtubule organization, a mitotic process that connects the spindle with the cell cortex to enable spindle positioning and the coordination of cytokinesis [55]. In line with this, tankyrase localizes to centrosomes and spindle poles [5,19,48,49], and silencing of tankyrase leads to abnormal spindles [49] as well as mitotic centrosome amplification and mis-localization [19]. Additionally, tankyrase was shown to associate with and PARylate NUMA1 in mitosis [30,49,50]. Silencing of NUMA1 displaces tankyrase from mitotic spindle poles, indicating that NUMA1 is an important recruiter of tankyrase [50]. We observe that NUMA1 TBM phosphorylation at position eight increases its affinity for TNKS2 ARC4, revealing a potential phospho-regulatory mechanism that controls tankyrase localization to mitotic spindle poles. Considering that tankyrase has many PARylation targets at the centrosome [19,28,48–50,56] and PAR is enriched at spindle poles [57], we propose that TBM position-eight phosphorylation could cluster tankyrase at centrosomes, followed by allosteric activation [25,45] and substrate PARylation, which in turn is required for proper centrosome function.

## 4. Conclusion

In this study, we present the first evidence that a subset of tankyrase-effector complexes is regulated by phosphorylation of TBMs at position eight. Our *in silico* analysis will aid the discovery of novel tankyrase targets regulated in this manner (electronic supplementary material, table S1). These observations illustrate how distinct post-translational modifications are interconnected, creating multi-tiered regulatory networks with multiple layers of control.

### 4.1. Limitations of the study

Our study is limited to phosphorylated peptides rather than full-length proteins, and to a single tankyrase ARC (TNKS2 ARC4) rather than the complete effector recruitment platform (ARCs 1−5 of both tankyrase paralogues). Despite these limitations, our biophysical, structural, *in silico* and phylogenetic analyses point towards a novel mechanism of modulated effector recruitment to tankyrase that should be explored more extensively. While we identify candidate kinases for MDC1 TBM1 S8 phosphorylation in cells, we did not explore the biological impact of the phosphorylation on downstream signalling, which could be addressed in future studies.

## 5. Material and methods

### 5.1. Protein expression

Human TNKS2 ARC4 and the K604A mutant variant were expressed and purified as described previously [28,58]. BL21-CodonPlus(DE3)-RIL *Escherichia coli* cells were transformed with the pETM30-2-TNKS2 ARC4 (residues 488−649; NM_025235.2) construct containing a cleavable hexahistidine-GST tag. A single colony was selected and expanded in LB media (100 ml) with 50 µg ml^−1^ kanamycin overnight. Subsequently, this culture was used to inoculate Terrific Broth (TB) supplemented with kanamycin (20 ml starter culture per litre of TB media). Cultures were grown at 37°C with shaking (180 r.p.m.) to an optical density (OD_600_) of 2.0. Isopropyl-β-D-1-thiogalactopyranoside (IPTG) (0.5 mM) was added to induce protein expression, and the cultures were incubated at 18°C overnight. The next day, cells were pelleted by centrifugation at 4000*g*, for 20 min. The pellet was stored at −80°C until purification.

### 5.2. Protein purification

The cell pellet was resuspended in lysis buffer (50 mM Tris-HCl pH 7.5, 500 mM NaCl, 10 mM imidazole, 5 mM β-mercaptoethanol) containing lysozyme (100 µg ml^−1^) and Pierce protease inhibitor cocktail tablets (1 per 50 ml lysis buffer). Cells were lysed by sonication on ice (5 min total sonication, 30% output level, 2 s on, 2 s off) using a Vibra-Cell sonicator (Sonics & Materials). Insoluble cell debris was removed by centrifugation (20 000*g*, 45 min) at 4°C. A 5 ml HisTrap affinity column (Cytiva) was equilibrated in lysis buffer, followed by binding of the recombinant protein from the cell lysate using an AKTA Start chromatography system. The column was washed with 50 column volumes (CVs) of lysis buffer before being connected to an AKTA Purifier chromatography system. The protein was eluted with a linear imidazole gradient run at 1 ml min^–1^: 0–100% elution buffer (50 mM Tris-HCl pH 7.5, 500 mM NaCl, 250 mM imidazole, 5 mM β-mercaptoethanol) over 99 ml. Protein-containing fractions were combined with tobacco etch virus (TEV) protease (1 : 50 weight ratio) to cleave the hexahistidine-GST tag while dialysing against a buffer containing 25 mM HEPES-NaOH pH 7.5, 100 mM NaCl, 5 mM β-mercaptoethanol. The protein was filtered through a 5 µm filter and then passed over a HisTrap affinity column to remove the tag and contaminants. The flow-through was then concentrated using a Vivaspin concentrator with a molecular weight cut-off (MWCO) of 10 000 Da (Sartorius) to a volume of less than 6 ml. The concentrated protein was further purified by gel filtration chromatography using a 120 ml Superdex 75 Hiload 16/600 column in gel filtration buffer (25 mM HEPES-NaOH, 100 mM NaCl, 2 mM TCEP, pH 7.5; 1.4 CV). Subsequently, fractions containing the target protein at the desired purity, as determined by SDS-PAGE analysis, were combined and concentrated. The concentrated protein was then flash-frozen in liquid nitrogen and stored at −80°C. Size exclusion chromatography with multi-angle light scattering (SEC-MALS) analysis showed that TNKS2 ARC4 is monomeric.

### 5.3. Peptides

Fluorescently labelled peptides for 3BP2 (FITC-βAla-LPHLQRSPPDGQSFRS-NH_2_), NUMA1 (FITC-βAla-TSKLPRTQPDGTSVPG-NH_2_) and FNBP1 (FITC-βAla-NCAQDRESPDGSYTEE-NH_2_) and their phosphorylated variants were synthesized and purified by Dr Gerald Gish (Lunenfeld-Tanenbaum Research Institute, Toronto, Canada). The fluorescently labelled MDC1 peptide (5(6)-carboxyfluorescein-βAla-QRGEPEGGSQ-NH_2_), its phosphorylated variants, and the corresponding non-binding G6R control peptide, were synthesized by JPT (Berlin, Germany) as crude stocks (greater than 70% pure by HPLC), with capping after each amino acid conjugation step to minimize accumulation and labelling of truncated peptides. Peptide stocks were quantified spectrophotometrically from suitable dilutions using a molar extinction coefficient of ε_492_ = 83 000 M^−1^ cm^−1^. The MDC1 peptide (acetyl-^943^ERDTQRGEPEGG-pS-QDQ^958^-NH_2_) for co-crystallization with TNKS2 ARC4 was synthesized and HPLC-purified by JPT (Berlin, Germany), reconstituted in a buffer containing 25 mM HEPES-NaOH pH 7.5, 100 mM NaCl, 2 mM TCEP and quantified by amino acid analysis.

### 5.4. Fluorescence polarization assays

Experiments were performed in technical duplicate. For 3BP2, NUMA1 and FNBP1, FP experiments were set up as described [28], using a Biomek FX liquid handling system (Beckman Coulter) to mix the assay components, and a PHERAstar plate reader (BMG Labtech) to measure FITC fluorescence intensities. For MDC1, FP experiments were performed as previously reported, using a POLARstar Omega plate reader (BMG Labtech) [58]. FP values (in polarization units, P) were obtained using the formula FP = (F_parallel_ − F_perpendicular_)/(F_parallel_ + F_perpendicular_) and converted to millipolarization units (mP). F_parallel_ and F_perpendicular_ refer to fluorescence intensities parallel and perpendicular to the excitation plane, respectively. Data were exported and analysed in GraphPad Prism (version 10). For FP experiments with *n* = 1 (3BP2, NUMA1, FNBP), technical duplicates were not averaged. FP values were baseline-corrected by subtracting the average FP values of corresponding no-protein wells from technical duplicate FP values to obtain ΔFP values, which were displayed individually. For experiments where *n* = 3 (MDC1), technical duplicates were averaged. Baseline correction was performed individually for each separate experiment, and data points presented as mean ± standard error of the mean (s.e.m.). Nonlinear regression analyses were performed in GraphPad Prism (version 10), handling ΔFP values separately, using a one-site total binding model to derive dissociation constants (*K*_*d*_) with standard errors of the fit.

### 5.5. Crystallization and protein structure determination

TNKS2 ARC4 at 20 mg ml^−1^ was mixed with a 1.5-fold molar excess of human phospho-MDC1 peptide encompassing residues 943−958, in 25 mM HEPES-NaOH pH 7.5, 100 mM NaCl, 2 mM TCEP. Crystallization was set up in 96-well sitting-drop vapour diffusion format using an SPT Labtech Mosquito liquid handler. Needle crystals grew within one month at 4°C upon mixing 150 nl of protein-peptide complex with 150 nl of crystallization solution (0.1 M Na cacodylate pH 6.5, 0.2 M Na acetate, 30% PEG 8000, from the QIAGEN Classics Suite). Crystals were cryoprotected in crystallization solution supplemented with 18% glycerol. X-ray diffraction data were collected at the Diamond Light Source on beamline I04, processed and scaled using XDS (v. 01 May 2016) [59], and merged using AIMLESS (v. 0.5.1), as implemented in the CCP4 package [60]. The high-resolution cut-off was chosen as described by Karplus and Diederichs, guided by a half-dataset correlation coefficient CC_1/2_ of 0.3 [61]. (Note that this gives rise to high *R*_merge_ and *R*_meas_ values reported in table 1.) The structure was determined by molecular replacement using PHASER (v. 2.7.15) [62], as implemented in the PHENIX package [63], with apo-TNKS2 ARC4 (PDB: 3TWQ, chain A, residues 488−644) [28] as a search model. The TNKS2 ARC4-MDC1 phospho-TBM1 model, with two molecules in the asymmetric unit, was generated by iterations of model building in Coot [64] and refinement in PHENIX Refine [65]. Side chain atoms not accounted for by electron density were deleted from the model. Structural representations were generated using UCSF Chimera [66], developed by the Resource for Biocomputing, Visualization, and Informatics at the University of California, San Francisco, with support from NIH P41-GM103311, and UCSF ChimeraX [67], developed by the same group, with support from National Institutes of Health R01-GM129325 and the Office of Cyber Infrastructure and Computational Biology, National Institute of Allergy and Infectious Diseases. For structural representations of the TNKS2 ARC4-MDC1 phospho-TBM1 complex, chains B and D are shown. For structural representations of the TNKS2 ARC4-3BP2 TBM complex (PDB 3TWR) [28], chains D and H are shown.

### 5.6. Ankyrin repeat cluster conservation analysis

Protein sequences of tankyrase homologues (orthologues and paralogues) were aligned using Clustal Omega [68], provided through the EMBL-EBI Web Services (v. January 2021) [69]. The alignment was reported previously [45]. National Center for Biotechnology Information (NCBI) accession numbers for tankyrase orthologues and paralogues included in this alignment are NP_003738.2 (*Homo sapiens*, human), NP_780300.2 (*Mus musculus*, house mouse), NP_989671.1 (*Gallus gallus*, red junglefowl), XP_035410112.1 (*Cygnus atratus*, black swan), XP_019389515.1 (*Crocodylus porosus*, saltwater crocodile), XP_037752450.1 (*Chelonia mydas*, green sea turtle), XP_026528659.1 (*Notechis scutatus*, tiger snake), XP_018099067.1 (*Xenopus laevis*, African clawed frog), XP_005451454 (*Oreochromis niloticus*, Nile tilapia), ENSDART00000111694.5 (*Danio rerio*, zebrafish), XP_005171802.1 (*Danio rerio*, zebrafish), XP_014351007.1 (*Latimeria chalumnae*, West Indian Ocean coelacanth), XP_036384182.1 (*Megalops cyprinoides*, Indo-Pacific tarpon), XP_032873798.1 (*Amblyraja radiata*, thorny skate), NP_079511.1 (*Homo sapiens*, human), NP_001157107.1 (*Mus musculus*, house mouse), NP_989672.1 (*Gallus gallus*, red junglefowl), XP_035404219.1 (*Cygnus atratus*, black swan), XP_019411065.1 (*Crocodylus porosus*, saltwater crocodile), XP_007059469.2 (*Chelonia mydas*, green sea turtle), XP_026526558.1 (*Notechis scutatus*, tiger snake), XP_018082988.1 (*Xenopus laevis*, African clawed frog), XP_005471626.1 (*Oreochromis niloticus*, Nile tilapia), XP_006006371.1 (*Latimeria chalumnae*, West Indian Ocean coelacanth), XP_036385759.1 (*Megalops cyprinoides*, Indo-Pacific tarpon), XP_032889463.1 (*Amblyraja radiata*, thorny skate), XP_020371197.1 (*Rhincodon typus*, whale shark), XP_032806710.1 (*Petromyzon marinus*, sea lamprey), XP_002121662.3 (*Ciona intestinalis*, sea vase), XP_019641281.1 (*Branchiostoma belcheri*, lancelet), XP_789260.4 (*Strongylocentrotus purpuratus*, Pacific purple sea urchin), XP_022094330.1 (*Acanthaster planci*, crown-of-thorns starfish), NP_651410.1 (*Drosophila melanogaster*, fruit fly), XP_321116.5 (*Anopheles gambiae*, African malaria mosquito), XP_032783964.1 (*Daphnia magna*, large water flea), XP_023333893.1 (*Eurytemora affinis*, marine copepod), XP_029842287.1 (*Ixodes scapularis*, deer tick), GBM18725.1 (*Araneus ventricosus*, ventricilous orbweaver spider), XP_022240762.1 (*Limulus polyphemus*, Atlantic horseshoe crab), CAD5118347.1 (*Dimorphilus gyrociliatus*, meiobenthic segmented worm), XP_005099438.1 (*Aplysia californica*, California sea hare), XP_022340347.1 (*Crassostrea virginica*, eastern oyster), KRZ50196.1 (*Trichinella nativa*, trichinella worm), KHN72016.1 (*Toxocara canis*, dog roundworm), CDS23197.1 (*Echinococcus granulosus*, hydatid worm), TNN12026.1 (*Schistosoma japonicum*, Japanese blood fluke), XP_012563232.1 (*Hydra vulgaris*, common hydra) and XP_019848937.1 (*Amphimedon queenslandica*, Great Barrier Reef sponge), as reported previously [45]. Percentage sequence identity was mapped onto an AlphaFold2 model of human TNKS obtained from the AlphaFold2 Protein Structure Database (accession number AF-O95271-F1, for comprehensive mapping onto a single tankyrase paralogue) [23,24], using UCSF Chimera [66]. Amino acid identity from 100 to 95% is indicated in red, followed by a linear gradient from red to white from 95 to 90%, and rendered white where the identity was less than 90%.

### 5.7. MDC1 conservation analysis

The protein sequences of MDC1 orthologues were retrieved from the Ensembl database using its REST API [70]. The sequences were aligned using Clustal Omega [68], through the EMBL-EBI Web Services (v. July 2024) [69]. The alignment was visualized in Jalview (v. 2.11.3.3) [71]. The Ensembl IDs for MDC1 orthologs were ENSP00000365588.3 (*Homo sapiens*, human), ENSPTRG00000017938 (*Pan troglodytes*, chimpanzee), ENSCSAG00000008273 (*Chlorocebus sabaeus,* green monkey), ENSCHIG00000014815 (*Capra hircus*, domestic goat), ENSCAFG00845007833 (*Canis lupus familiaris*, dog), ENSUMAG00000019676 (*Ursus maritimus*, polar bear), ENSCDRG00005018194 (*Camelus dromedarius,* camel), ENSAMEG00000013188 (*Ailuropoda melanoleuca*, giant panda) ENSPCTG00005009708 (*Physeter catodon*, sperm whale), ENSMSIG00000015149 (*Mus spicilegus*, steppe mouse), ENSDORG00000003207 (*Dipodomys ordii,* Ord’s kangaroo rat) ENSNGAG00000005054 (*Nannospalax galili*, Middle-East blind mole-rat) ENSSVLG00005006421(*Sciurus vulgaris*, red squirrel) ENSTMTG00000012925 (*Terrapene carolina triunguis*, turtle) ENSGEVG00005009939 (*Gopherus evgoodei*, Goode’s thornscrub tortoise) ENSNSUG00000019570 (*Notechis scutatus*, tiger snake) ENSCARG00000040270 (*Carassius auratus*, goldfish) ENSSTUG00000015500 (*Salmo trutta*, brown trout) [70].

### 5.8. Kinase prediction

The human MDC1 protein sequence ^944^EPEGGpS*QDQKGQ^960^ was searched in the Kinase Library [43] for the prediction of kinases phosphorylating MDC1 TBM1 at position eight. This resource generates prediction scores based on experimentally derived position-specific scoring matrices (PSSMs) for 303 serine/threonine kinases [43]. Results for the 10 top-scoring kinases were extracted and ranked by log2 score. These kinases were GRK7 (G-Protein-Coupled Receptor Kinase 7), DNAPK (DNA-Dependent Protein Kinase), ATM (Ataxia Telangiectasia Mutated), COT (Cancer Osaka Thyroid), CLK3 (CDC-Like Kinase 3), ATR (Ataxia Telangiectasia and Rad3-Related Protein Kinase), GSK3A (Glycogen Synthase Kinase 3 Alpha), PLK2 (Polo-Like Kinase 2), TLK2 (Tousled-Like Kinase 2), MTOR (Mechanistic Target of Rapamycin) [43]. This encompassed the following kinase groups: AGC (Protein kinase A, G and C families), PIKK (Phosphatidylinositol 3-kinase-related kinase family), STE (Sterile 20-like Serine/Threonine Kinases), and CMGC (CDK, MAPK, GSK3 and CLK kinases) [43]. Logo sequences for ATM and ATR kinases were built from their PSSM in the Kinase Library [43].

### 5.9. *In silico* prediction of tankyrase-binding motif position-eight serine/threonine/tyrosine phosphorylation and gene ontology biological processes enrichment analysis

We started with a previously filtered dataset of 11 698 predicted human octapeptide TBMs [28]. Briefly, this dataset includes peptides scored for their agreement with an experimentally derived TBM consensus, expressed via a PSSM [28]. The list used for our analysis here included peptides with a minimal conformance to the TBM consensus (tankyrase targeting score (TTS) ≥ 0.385). Unlikely candidates, based on topology and low relative motif disorder, were removed [28]. We then selected all peptides which contain a serine, threonine or tyrosine at position eight in the motif and searched these sequences on PhosphoSitePlus [44] using the protein sequence search function. We then extracted these data to obtain information on any modification occurring in these sequences and mapped octapeptides back to their host proteins using the associated UniProt IDs [72]. The data were filtered for phosphorylation events that appeared at position eight in the motif and split into serine (pS8), threonine (pT8) and tyrosine (pY8) arms. GO enrichment analysis (date: 23 October 2023) [46] was performed using the associated UniProt IDs [72]. Data were wrangled in R and plots generated using the ggplot2 package (v. 3.3.3; [73]).

## 6. STRING analysis

The following proteins (by UniProt ID) represented the four most highly enriched GOs for predicted tankyrase effectors with TBM serine/threonine phosphorylation at position eight: establishment of centrosome localization (Q8IVT2, Q12959, P15311, Q8TEW8, Q8TEW0); centrosome localization (Q8TD16, Q8IVT2, Q12959, Q9UH99, P49792, P15311, Q8TEW8, Q8TEW0); microtubule organization centre localization (Q8TD16, Q8IVT2, Q12959, Q9UH99, P49792, P15311, Q8TEW8, Q8TEW0) and astral microtubule organization (Q7Z460, Q14980, Q12959, P15311) [72]. These were collected and duplicates removed for subsequent input into STRING pathway analysis using the STRING database (version 11.5) [74].

## Data Availability

X-ray crystallography data and model coordinates have been deposited to the Protein Data Bank (PDB) under accession number 9QJZ. Raw data associated with figure 2 (FP), figure 3/electronic supplementary material, table S1 (TTS ranking filtered by amino acid at position eight, code for retrieval of post-translational modifications from PhosphoSitePlus), figure 4 (FP) and electronic supplementary material, figure S2 (FP) are available through Mendeley Data [75]. Data and relevant code used in this work to retrieve post-translational modifications for each peptide sequence are stored in GitHub: https://github.com/benbroadway/PhosphoSitePlus-Peptide-Search and have been archived within the Zenodo repository [76]. Supplementary material is available online [77].
